# Carnosine-Enriched Chicken Meat Improves Microvascular Function and Anti-Inflammatory Phenotype in Patients with Chronic Coronary Syndrome

**DOI:** 10.3390/nu18060928

**Published:** 2026-03-15

**Authors:** Dora Uršić, Nikolina Kolobarić, Ines Drenjančević, Zrinka Mihaljević, Petar Šušnjara, Ana Stupin, Ivana Jukić, Aleksandar Kibel

**Affiliations:** 1 Department of Rheumatology, Clinical Immunology and Allergology, University Hospital Center Osijek, 31000Osijek, Croatia; dora.cvitkusic@gmail.com; 2 Department of Internal Medicine and History of Medicine, Faculty of Medicine Osijek, Josip Juraj Strossmayer University of Osijek, 31000 Osijek, Croatia; 3 Scientific Centre of Excellence for Personalized Health Care, Josip Juraj Strossmayer University of Osijek, 31000 Osijek, Croatia; nbdujmusic@mefos.hr (N.K.); ines.drenjancevic@mefos.hr (I.D.); zmihaljevic@mefos.hr (Z.M.); psusnjara@kifos.hr (P.Š.); ana.stupin@mefos.hr (A.S.); 4 Department of Physiology and Immunology, Faculty of Medicine Osijek, Josip Juraj Strossmayer University of Osijek, 31000 Osijek, Croatia; 5 Department of Interdisciplinary Sciences, Faculty of Kinesiology Osijek, Josip Juraj Strossmayer University of Osijek, 31000 Osijek, Croatia; 6 Scientific Unit for Medical Research, International Medical Center Priora, 31431 Cepin, Croatia; 7 Department of Clinical Medicine, Faculty of Dental Medicine and Health, Josip Juraj Strossmayer University of Osijek, 31000 Osijek, Croatia

**Keywords:** blood pressure, carnosine, chronic coronary syndrome, endothelium, functional food, microcirculation

## Abstract

Background/Objectives: This study investigated the effect of the consumption of carnosine-enriched chicken meat on endothelium-dependent and independent microvascular reactivity and inflammatory mediators in patients with chronic coronary syndrome (CCS). Materials and Methods: In total, 38 CCS participants were randomized to two groups: the Control group (N = 19), who consumed regular chicken meat, and the Carnosine group (N = 19), who consumed carnosine-enriched chicken meat for 3 weeks. Skin microvascular reactivity in response to vascular occlusion (PORH), acetylcholine (ACh ID), sodium nitroprusside (SNP ID), and local thermal hyperemia (LTH) was measured. Arterial blood pressure (BP), heart rate (HR), biochemical parameters, anti- and proinflammatory cytokine levels, and markers of oxidative stress were assessed before and after the intervention. Results: The consumption of carnosine-enriched chicken meat improved endothelium-dependent (PORH, LTH) and endothelium-independent vasodilation (SNP ID). Systolic BP (SBP), diastolic BP (DBP), and mean BP (MAP), as well as serum concentrations of tumor necrosis factor-alpha (TNF-α), interleukin-6 (IL-6), and endoglin, decreased from the initial measurements. Conclusion: The consumption of carnosine-enriched chicken meat enhances microvascular endothelium-dependent and independent vasodilatation. Patients with CCS can benefit from carnosine-enriched chicken meat consumption through improved hemodynamic parameters, reduced inflammation, and enhanced microvascular relaxation.

## 1. Introduction

Cardiovascular disease continues to be the primary cause of mortality worldwide [1]. Endothelial dysfunction in cardiovascular disease pathogenesis arises from multiple factors including dyslipidemia, hypertension, proinflammatory cytokines, and oxidative stress [2,3]. Coronary artery disease (CAD) involves the development of atherosclerotic plaques within the epicardial coronary arteries. Depending on the process affecting the coronary arteries, patients can be classified as having acute or chronic coronary syndrome (CCS) [4]. Hypertension triggers CAD onset [5]. Endothelial dysfunction contributes to increased peripheral resistance by promoting vasoconstriction and vascular remodeling in resistance arteries, thereby facilitating the onset of hypertension [6]. Therapeutic strategies designed to lower BP while enhancing endothelial function help to reduce the impact of CAD [5,6].

Carnosine, a dipeptide composed of β-alanine and L-histidine, is endogenously synthesized and primarily accumulates in skeletal muscle, with substantial levels also present in brain, cardiac, and gastrointestinal (GI) tissues. Carnosine metabolism is a tightly regulated cycle of synthesis from dietary precursors, partial intact absorption, rapid serum degradation, and tissue-specific resynthesis. Carnosine synthase (CARNS1) catalyzes the ATP-dependent bonding of β-alanine—a rate-limiting precursor derived from meat dipeptides or liver breakdown of uracil/thymidine—with L-histidine. After consumption, a portion of ingested carnosine survives GI digestion intact and is absorbed due to relatively low carnosinase activity in the intestinal mucosa. Once in plasma, serum carnosinase (CN1) rapidly hydrolyzes carnosine to β-alanine and L-histidine, which are then recycled for carnosine resynthesis in muscle, brain glial cells, heart, kidneys, and liver [7,8]. Functioning as both an enzymatic-independent ROS quencher and an endogenous antioxidant, carnosine demonstrates inflammation-suppressing benefits [9,10], pH stabilization capacity [11], metal-chelating ability, and antiglycation properties [12,13]. Furthermore, it also reduces lipid peroxidation [13] and improves antioxidant capacity [14]. Carnosine inhibits the formation of advanced glycation end products, as measured by a fluorometric assay in animal models, potentially reducing oxidative stress and thereby decreasing vascular injury [15].

Because of carnosine’s positive impact on metabolism in various organs, efforts have been made to develop meat enriched with carnosine [16]. Animal studies have demonstrated that carnosine supplementation may lower BP and reduce serum lipids, thus inhibiting the progression of hypertension and atherosclerosis. In obese Zucker rats, administration of carnosine decreased serum lipids and creatinine and urea levels [17]. Triglyceride levels in the heart and liver, along with IL-6 and TNF-α levels, were significantly reduced following carnosine and histidine intake in Balb/c diabetic rats [18]. Carnosine supplementation, particularly alongside vitamin E, showed a protective effect against doxorubicin-induced cardiotoxicity in the rat myocardium [19]. In patients with chronic heart failure receiving carnosine treatment for six months, improvements were observed in a 6 min walk test distance [20]. Nonetheless, well-designed human clinical trials exploring carnosine’s impact on the vascular function are still limited [21].

The hypothesis of this study was that carnosine supplementation in the form of functional food could have a favorable effect on endothelial function and inflammation in patients with CCS. Therefore, the main aim was to evaluate the effects of carnosine-enriched chicken meat consumption on BP, microvascular reactivity, lipid profiles, biomarkers of oxidative stress, and levels of anti- and proinflammatory mediators in patients with CCS.

## 2. Materials and Methods

### 2.1. Study Design and Participants

**Figure 1:**
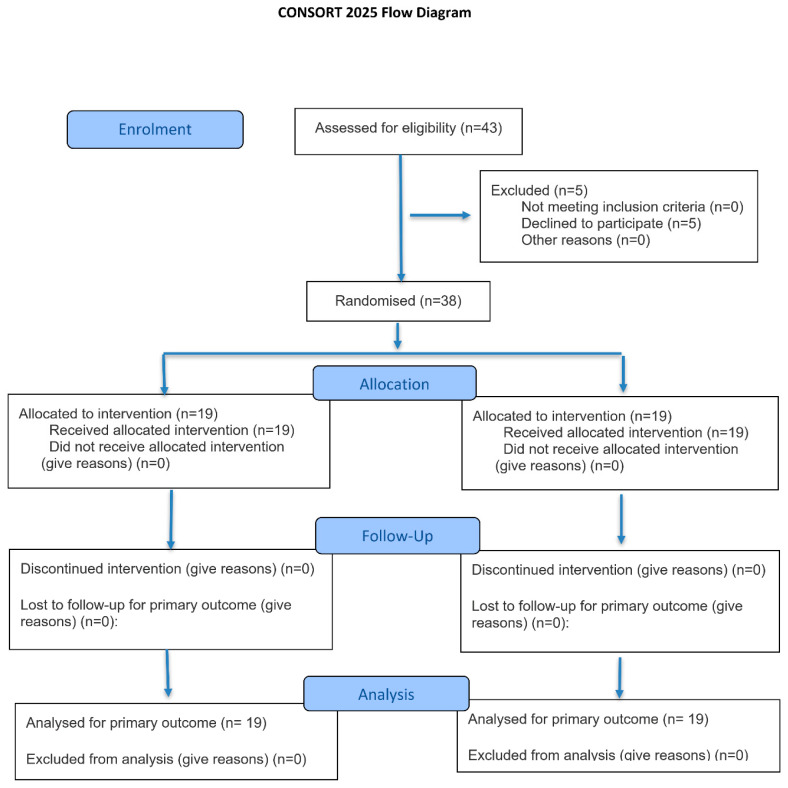
Consort 2025 flow diagram.

This double-blind, randomized trial involved 38 individuals (both sexes, aged ≥ 18 years) with established CCS. The diagnosis of CCS followed the criteria outlined in the European Society of Cardiology guidelines, including patients with previously confirmed, clinically stable CAD [22]. Recruitment was conducted at the Department of Cardiovascular Diseases, University Hospital Centre Osijek, Croatia, from April 2023 to July 2024. The study design and participant flow are summarized in Figure 1. Exclusion criteria were malignancy, inherited metabolic or autoimmune disorders, major surgery within 3 months, significant injuries within the last 6 months, neurodegenerative or cerebrovascular disease, post-cardiac arrest syndrome ≤ 3 months, use of immunomodulatory drugs (monoclonal antibodies, immunosuppressants, corticosteroids), anemia (Hb < 110/100 g/L for males/females), chronic pulmonary, hepatic, or renal disease, active or chronic infection such as tuberculosis, and active substance or alcohol abuse.

All participants signed written informed consent after complete study explanation. The investigation complied with the current Declaration of Helsinki and was approved by the Faculty of Medicine Osijek (No: 2158-61-46-23-19; 23 February 2023) and Osijek University Hospital (No: R2-3775/2022; 14 June 2022) Ethics Committees. The study forms part of the registered trial examining carnosine supplementation’s cardiovascular impacts (ClinicalTrials.gov identifier: NCT05723939; registered 1 February 2023).

### 2.2. Production of Carnosine-Enriched Chicken Meat

The production of carnosine-enriched chicken meat followed the method previously developed by researchers at the Faculty of Agrobiotechnical Sciences, Osijek, Croatia [23]. For this study, Ross 308 hybrid male broilers were used, and the fattening period lasted 42 days. For the first 21 days, the broilers were fed a standard base mixture. From days 22 to 42, they received either a standard pelleted finisher mixture (Control group) or a specially designed finisher mixture containing a combination of β-alanine and L-histidine with the addition of MgO. The broilers that consumed the standard finisher were used as regular chicken meat, while the broilers that consumed the designed composition finisher were used as carnosine-enriched chicken meat. The Control group did not receive β-alanine or L-histidine supplementation, either alone or in combination. Carnosine levels in chicken muscle tissue were analyzed specifically for the present study in five randomly selected broilers from each group by the same research team using high-performance liquid chromatography (HPLC) with the methodology of Kralik et al. [23]. Analysis showed higher carnosine levels in carnosine-enriched chicken meat (846.8 mg/kg in breast and 341.5 mg/kg in thigh) compared to the Control group (537.8 mg/kg and 167.7 mg/kg, respectively), consistent with a previous study by Kolobarić et al. [24].

### 2.3. Study Protocol

Random allocation of subjects into two groups was performed by a coin toss, with heads designating the experimental group and tails the control group; 19 subjects comprised the control group (Control group) and consumed regular chicken meat, and 19 subjects comprised the experimental group (Carnosine group) and consumed chicken meat enriched with carnosine. A unique study code that did not contain any personal data was assigned to each participant. The study employed double blinding, ensuring that neither participants nor investigators were aware of group assignment. Participant data were coded and numbered by an independent researcher without participant contact.

The dietary protocol lasted for 3 weeks. Each subject received 21 packages of chicken meat (10.5 kg in total) prepared for daily intake, as well as instructions for its preparation. Each package contained 500 g of fresh meat for daily use, approximately 350 g of chicken breast and 150 g of chicken thigh. Based on measurements of carnosine content in the chicken meat, it was estimated that the Control group consumed 410 mg of carnosine/day and the Carnosine group consumed 590 mg of carnosine/day. During the study, no other forms of carnosine supplementation (e.g., capsules) were taken. Participant compliance was supported through regular telephone follow-ups and guidance to record detailed dietary intake in personal diet diaries. These diaries were adapted from the format developed by Kolobarić et al. [25].

The study took place at the Laboratory for Clinical and Sports Physiology, Department of Physiology and Immunology, Faculty of Medicine, Osijek, Croatia. The protocol comprised two visits: all assessments occurred on day 1 and the day after protocol completion. Measurements were performed after an overnight fast, and participants were requested to avoid vigorous activity in the previous 24 h.

### 2.4. Anthropometric and Hemodynamic Parameters

Participant height, weight, waist circumference, and hip measurements facilitated calculation of body mass index (BMI) and waist-to-hip ratio (WHR). At both visits, HR and BP were obtained as the average of three readings taken after 15 min seated rest using an automated oscillometric device (OMRON M3, OMRON Healthcare Inc., Osaka, Japan).

An impedance cardiography (ICG) system (CardioScreen 2000 Professional, Medizinische Messtechnik GmbH, Ilmenau, Germany) provided assessment of systemic hemodynamics by measuring thoracic electrical conductivity fluctuations and processing cardiodynamic parameters continuously. Four sensors positioned on the neck and chest enabled ICG to capture impedance changes from blood volume fluctuations and thoracic fluid shifts in participants. The system also recorded thoracic electrical potential variations, producing signals resembling electrocardiograms from atypical leads. Using the device’s original software, hemodynamic parameters were obtained directly, via calculation, or normalized to body surface area (BSA), including HR, stroke volume (SV), cardiac output (CO), cardiac index (CI), systemic vascular resistance index (SVRI), and total arterial compliance index (TACI).

To measure heart rate variability (HRV), the Heart Rhythm Scanner device (Biocom 4000, Poulsbo, Washington, USA) was used, which records ECG signals in the supine position over a period of 5 min. Measurements were conducted in a room with a constant temperature of 23.5 °C (standard deviation 0.5 °C), between 8:30 and 12:00 a.m.

### 2.5. Biochemical Marker Analysis

Venous blood specimens were obtained and evaluated for complete blood count, renal function markers, electrolytes, liver function tests, high-sensitivity C-reactive protein (hsCRP), blood glucose, urate, total cholesterol, low-density lipoprotein (LDL), high-density lipoprotein (HDL), triglycerides. All testing was conducted using routine laboratory procedures at the Department of Clinical Laboratory Diagnostics, University Hospital Center Osijek, Croatia.

### 2.6. Microvascular Endothelium-Dependent and Independent Vasodilatation

Microvascular reactivity was evaluated using laser Doppler flowmetry (MoorVMS-LDF, Axminster, UK). Microvascular assessments included post-occlusive hyperemia (PORH), skin perfusion responses to acetylcholine (ACh; endothelium-dependent vasodilator), sodium nitroprusside (SNP; endothelium-independent vasodilator), and local thermal hyperemia (LTH). Participants reclined in a temperature-controlled room (23.5 ± 0.5 °C) for 30 min prior to assessment. A laser Doppler probe (13–15 cm length) was positioned on the proximal left volar forearm. After establishing a 5 min baseline blood flow, brachial artery occlusion was induced for 1 min using a pneumatic upper arm cuff raised to 30–50 mmHg exceeding systolic. Following cuff release to elicit PORH, measurements continued for 2 additional minutes. Measurements captured microcirculatory blood flow variations throughout the occlusion-release sequence. Expressed in perfusion units (PU), the data underwent software analysis to compute the area under the curve (AUC), resulting in baseline-adjusted percentage flow shift from occlusion to reperfusion (R–O%) [26].

Post-PORH, iontophoresis combined with LDF administered ACh and SNP. Baseline microvascular blood flow was established for 5 min, followed by iontophoretic delivery of positively charged ACh (1%) or negatively charged SNP (1%) to elicit maximal stable LDF plateaus. Microcirculatory flow in PU was quantified, with AUC determined by software across baseline and vasodilator application [27].

Nitric oxide (NO)-dependent endothelial vasodilation in skin microcirculation was assessed via LTH response measured by LDF. Local heating induces a biphasic skin blood flow increase: an initial rapid peak within 90–120 s via sensory nerve axon reflex, followed by a sustained plateau after 30 min primarily mediated by endothelial NO (contributing 60–70% of plateau response). Following 5 min baseline flow, skin temperature was raised from baseline to 42 °C at 0.1 °C·s^−1^ and held constant using a local heater (moorVMS-HEAT, Moor Instruments, Axminster, UK) [28]. Blood flow was monitored continuously until plateau stabilization (30–45 min post-heating). Microcirculatory flow in PU underwent software analysis to compute AUC for baseline and sustained heating plateau phases. Results quantified the heating-induced flow increase relative to the baseline.

### 2.7. Oxidative Stress Biomarker and Antioxidant Capacity

The thiobarbituric acid-reactive substance (TBARS) assay quantified serum malondialdehyde (MDA), formed during polyunsaturated fatty acid lipid peroxidation. Therefore, the TBARS method serves as an indicator of lipid peroxidation. MDA reacts with thiobarbituric acid (TBA) to produce a pink chromogen (TBARS), detectable by absorbance measurement at 532 and 572 nm using a Nanophotometer (P300 UV/VIS, IMPLEN, Munich, Germany), with MDA standards expressed as µM [29].

The ferric-reducing ability of plasma (FRAP) assay quantifies antioxidant capacity. Antioxidants serve as reducing agents in FRAP, transforming Fe^3+^ into Fe^2+^ in acidic medium to generate the blue tripyridyltriazine-Fe^2+^ complex. Absorbance was measured at 593 nm using a Nanophotometer (P300 UV/VIS, IMPLEN) with Trolox standards (mM/L Trolox) [30].

### 2.8. Serum Concentrations of Anti- and Proinflammatory Cytokines and Adhesion Molecules

Serum concentrations of intercellular adhesion molecule 1 (ICAM-1), vascular cell adhesion molecule 1 (VCAM-1), E-selectin, endoglin, and monocyte chemoattractant protein-1 (MCP-1) were quantified alongside transforming growth factor beta 1 (TGF-β1), TNF-α, complement component 3a (C3a), interferon gamma (IFN-γ), IL-6, interleukin 10 (IL-10), interleukin 17A (IL-17A), and interleukin 23 (IL-23). ProcartaPlex Human TGF-β1 Simplex, ProcartaPlex Human C3a Simplex, and Human ProcartaPlex Mix&Match 7-plex magnetic bead panels on the Luminex 200 platform (Luminex Corporation, Austin, TX, USA) were used. Data analysis utilized ProcartaPlex Analyst software v1.0, reporting concentrations in pg/mL. The analysis was carried out at the Laboratory of Molecular and HLA Diagnostics, University Hospital Osijek, Croatia.

### 2.9. Statistical Analysis

Results are expressed as arithmetic mean and standard deviation or median with interquartile range, based on Shapiro–Wilk normality testing. Pre/post-protocol changes within Control and Carnosine groups were analyzed by paired *t*-tests for normally distributed data or Wilcoxon signed-rank tests for non-normal distributions. Baseline intergroup comparisons employed Student’s *t*-test or Mann–Whitney U test according to distribution normality. Post-intervention intergroup differences were analyzed by analysis of covariance (ANCOVA) using baseline values as covariates. Spearman’s rank test assessed correlations between paired datasets. Significance was set at α = 0.05 with 80% statistical power throughout; *p* ≤ 0.05 indicated statistical significance. GraphPad Prism v6.01 (GraphPad Software, San Diego, CA, USA) and SigmaPlot v16.0 (Systat Software, Chicago, IL, USA) performed all analyses.

## 3. Results

### 3.1. Anthropometric Characteristics of Patients with CCS

Anthropometric parameters (BMI, WHR) remained unchanged post-carnosine-enriched or regular chicken meat consumption compared to baseline measurements. Study groups showed no significant BMI or WHR differences at baseline or following dietary intervention (Table 1).

### 3.2. Hemodynamic Parameters and Heart Rate Variability Assessment

SBP, DBP, and MAP decreased substantially in the Carnosine group following intervention, alongside significantly reduced SBP and MAP compared to the Control group (adjusted for baseline). The Carnosine group showed higher baseline HR, followed by significant post-protocol reduction. The Carnosine group exhibited significant SV elevation after intervention relative to the Control group (adjusted for baseline). The dietary protocol did not significantly affect CO, CI, SVRI or TACI within or between the two study groups (Table 1). The values of heart rate variability parameters (mean RR, SDNN, RMSSD, pNN50, TI, TP, VLF, LF, HF, LF/HF) did not significantly change within or between groups after the protocol (Table 2).

### 3.3. Blood Profile and Biochemical Marker Analysis

The obtained values of blood count parameters in both study groups of patients with CCS were within reference ranges. After the dietary protocol, there were no significant differences within the study groups in the blood count parameters, except for the value of mean corpuscular hemoglobin (MCH), which significantly increased in the Carnosine group (Table 3).

There were no differences between the groups in any of the measured blood count and biochemical parameters at baseline and post-intervention. Although urea concentration significantly increased within the Carnosine group, it remained within the reference interval. Serum sodium levels were within the reference range in both study groups; however, a significant decrease was observed in the Control group after the intervention (Table 4).

### 3.4. Measurements of Blood Flow and Vascular Reactivity in the Skin Microcirculation of Patients with CCS

**Figure 2:**
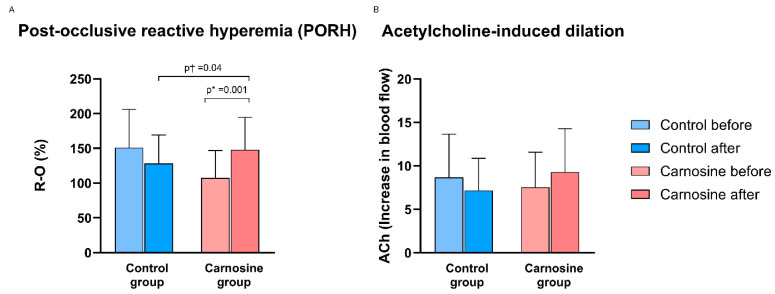
The effect of consumption of regular and carnosine-enriched chicken meat on post-occlusive reactive hyperemia (PORH) (**A**) and acetylcholine-induced dilation (ACh ID) (**B**) values in patients with CCS. Results are expressed as mean (standard deviation). *p* * ≤ 0.05 (paired *t*-test); *p* † ≤ 0.05 (ANCOVA, baseline covariate).

PORH values were significantly lower in the Carnosine group compared to the Control group at the baseline. PORH values significantly increased within the Carnosine group post-protocol and relative to the Control group (adjusted for baseline) (Figure 2A). There was no significant change in ACh-induced dilation (ACh ID) after the consumption of regular and carnosine-enriched chicken meat compared with the baseline measurements or between the groups (Figure 2B).

**Figure 3:**
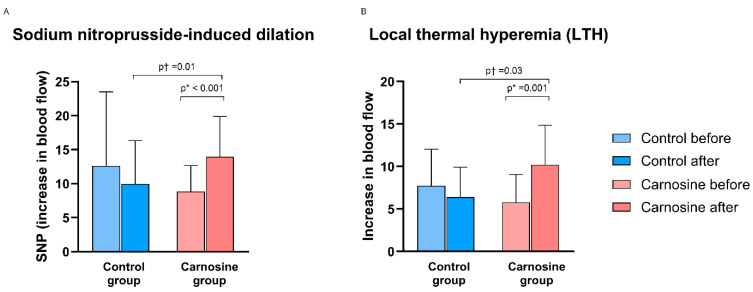
The effect of consumption of regular and carnosine-enriched chicken meat on sodium nitroprusside-induced dilation (SNP ID) (**A**) and local thermal hyperemia (LTH) (**B**) values in patients with CCS. Results are expressed as mean (standard deviation). *p* * ≤ 0.05 (paired *t*-test); *p* † ≤ 0.05 (ANCOVA, baseline covariate).

SNP-induced dilation (SNP ID) and LTH significantly increased within the Carnosine group post-protocol and relative to the Control group (adjusted for baseline) (Figure 3A,B).

### 3.5. Oxidative Stress Biomarker and Antioxidant Capacity

**Figure 4:**
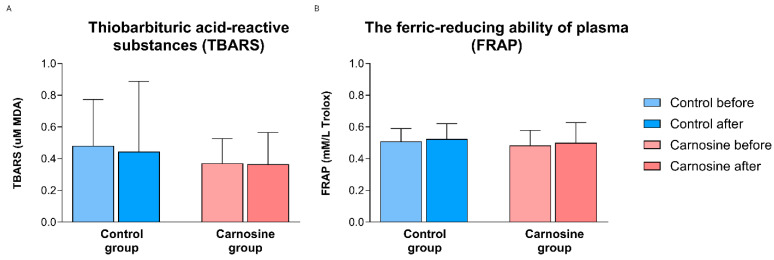
The effect of consumption of regular and carnosine-enriched chicken meat on oxidative stress biomarker (TBARS) (**A**) and antioxidant capacity (FRAP) (**B**) values in patients with CCS. Results are expressed as mean (standard deviation).

TBARS and FRAP remained unchanged after regular and carnosine-enriched chicken consumption versus baseline within or between groups (Figure 4A,B).

### 3.6. Serum Concentrations of Anti- and Proinflammatory Cytokines and Adhesion Molecules

Within the Control group, serum cytokines and chemokines remained unchanged after regular chicken meat consumption, whereas the Carnosine group showed significant TNF-α and IL-6 declines. Endoglin levels decreased markedly in the Carnosine versus Control group post-dietary protocol (adjusted for baseline) (Table 5).

### 3.7. Correlations

An analysis of associations between blood flow measurements, serum concentrations of cytokines, adhesion molecules, and hemodynamic parameters was performed. In the Carnosine group, SNP ID moderately inversely correlated with ICAM-1 values (r = −0.323, *p* = 0.05), as well as with IFN-γ values (r = −0.368, *p* = 0.03). Also, in the Carnosine group, a moderate inverse correlation between PORH and SBP values (r = −0.316, *p* = 0.05), and between LTH and MAP values was observed (r = −0.333, *p* = 0.04). In the Control group, ACh ID moderately directly correlated with DBP (r = 0.340, *p* = 0.04).

## 4. Discussion

In this study, we explored the effect of carnosine-enriched food on microvascular function in patients with CCS. Our key findings demonstrate that (a) the consumption of carnosine-enriched chicken meat significantly reduced SBP, DBP and MAP in participants with CCS; (b) the consumption of carnosine-enriched chicken meat improved endothelium-dependent (PORH, LTH) and endothelium-independent vasodilation (SNP ID) in the microcirculation of participants with CCS; (c) the consumption of carnosine-enriched chicken meat reduced serum concentrations of proinflammatory cytokines TNF-α and IL-6 and marker of endothelial activation, endoglin; (d) the consumption of carnosine-enriched chicken meat showed no effect on oxidative stress and antioxidant capacity (TBARS, FRAP). No prior research has investigated whether carnosine supplementation in the form of a functional food affects microvascular reactivity in participants with CCS. Carnosine-enriched chicken meat was well-tolerated and there were no significant side effects.

Carnosine supplementation lowered BP in animal models. Indeed, in hypertensive DOCA–salt rats, carnosine supplementation attenuated SBP elevation during hypertension [31]. Also, 250 mg/kg daily intraperitoneal intake of carnosine for 16 weeks reduced abdominal obesity and blood pressure in rats on a high-fat–carbohydrate diet [32]. Mechanisms underlying carnosine’s antihypertensive effect include the histamine/histidine pathway [33], NO/cGMP signaling [34], and autonomic nervous system modulation [35]. Human data on carnosine supplementation’s effects on hemodynamic parameters remain scarce. Saadati et al. found no BP changes after 14 weeks of 2 g daily carnosine in prediabetes and type 2 diabetes mellitus adults [36]. However, Peric et al. demonstrated that carnosine-enriched chicken meat significantly reduced DBP and MAP in healthy male competitive athletes, indicating a possible BP-lowering effect of carnosine even in normotensive individuals [37]. Our participants in both groups were hypertensive at baseline, but a significant reduction in SBP, DBP, and MAP was only seen in the Carnosine group.

In our study, HR declined markedly in the Carnosine group relative to the Control group post-intervention. HR levels were significantly decreased in a Carnosine group after 13 weeks of chicken extract (40% carnosine and anserine) supplementation in elderly participants [38]. As previously reported, Carnosine’s HR-lowering effect may involve elevated intracellular Ca^2+^ and improved cardiomyocyte contractility [39]. Findings from a study in young women demonstrated that chicken extract (carnosine-rich) decreased HRV [40]. On the contrary, no effect on HRV was observed in our study.

Carnosine enhances cardiac contractility in a concentration-dependent manner in healthy rat and pig hearts [41]. However, carnosine supplementation for 2 weeks did not improve contractility in an isolated rat heart exposed to hypoxia and reoxygenation [42]. In our study, SV significantly increased in the Carnosine group post-protocol compared to the Control group, indicating a possible positive effect of carnosine in improving heart contractility in CCS patients.

In animal studies, carnosine showed a positive influence on the lipid profile. In type 2 diabetes rat models, carnosine lowered serum lipid levels [17,43]. In a previously mentioned study, carnosine supplementation in high-fat, high-carbohydrate-diet-fed rats normalized total cholesterol and LDL levels [32]. Here, we observed no alterations in lipid profile following carnosine-enriched chicken meat consumption. This could reflect predominantly normal baseline serum lipid concentrations.

Carnosine has a diverse effect across endothelial cells of various blood vessels. While carnosine causes endothelium-independent vasodilatation in isolated rat aorta [34], in the rabbit saphenous vein, it provokes a vasoconstrictive effect [44]. This suggests that carnosine displays differential effects across tissue beds. The present study reveals that consumption of carnosine-enriched food markedly enhances endothelium-dependent and -independent microvascular vasodilation in CCS patients. On the other hand, in patients with prediabetes and type 2 diabetes mellitus, carnosine failed to improve endothelial dysfunction measures [36]. These findings can be attributed to the study population, as most of the patients had normal cardiovascular parameters at baseline. However, a previously mentioned study by our group, involving healthy individuals consuming carnosine-enriched chicken meat over 3 weeks, showed improved PORH and SNP ID [37].

Endothelial viability depends on NO. The effects of carnosine on NO synthesis vary from inhibitory to stimulatory across different vascular beds [21,45,46]. In our study, the intervention led to substantial LTH enhancement in the Carnosine group. LTH is a biphasic process; the initial response is neurogenic (axon reflex), while the sustained increase in blood flow is largely mediated by endothelial NO production. The inverse association of PORH response with SBP, as well as LTH response with MAP values, suggests that the carnosine blood pressure-lowering effect could be related to induced endothelium-dependent changes.

In aged rats, carnosine (250 mg/kg/day) significantly reduced oxidative stress, but not in young rats [47]. In healthy athletes, carnosine-enriched chicken meat significantly decreased lipid peroxidation and superoxide production while increasing serum enzyme activity [24]. Surprisingly, oxidative stress and antioxidant capacity remained unchanged within and across CCS patient groups.

A relatively small sample size represents a limitation of this study; however, despite this, the results clearly indicate the observed effects of the intervention. A key finding of this study is reduced TNF-α and IL-6 levels after carnosine-enriched chicken meat consumption. TNF-α, a primary proinflammatory cytokine, sustains low-grade systemic inflammation. Its proatherogenic actions on the endothelium involve promoting ROS production, diminishing NO bioavailability, and enhancing permeability [48]. Proinflammatory TNF-α signaling rapidly upregulates endothelial expression of E-selectin, VCAM-1, and ICAM-1, facilitating leukocyte recruitment and transmigration into the vascular wall. TNF-α signal transduction promotes cardiovascular impairment, atherogenesis, hypertension, and pathological remodeling after myocardial infarction [48,49]. As a polymorphic cytokine, IL-6 demonstrates both pro-atherosclerotic and protective roles on plaque formation and progression through vascular smooth muscle cell proliferation, endothelial activation, and platelet stimulation [50,51]. A dose of 1000 mg/kg/day carnosine for 1 month reduced TNF-α and IL-6 in rats [52]. Nevertheless, human trial findings are inconsistent. Carnosine supplementation lowered TNF-α but not IL-6 across nine trials with 350 participants [53].

Early aortic endothelial dysfunction features elevated endoglin levels [54], which correlate with total cholesterol and atherosclerosis progression in animal models [55]. Plasma endoglin levels were markedly elevated in patients with myocardial infarction [56]. No significant endoglin alterations occurred with carnosine use in healthy athletes [24]. However, the present study shows that endoglin levels were significantly decreased in the Carnosine group compared to the Control group after the dietary protocol, suggesting a favorable effect of carnosine on endothelial function in CCS patients.

## 5. Conclusions

The consumption of carnosine-enriched chicken meat by CCS patients improved endothelium-dependent (PORH, LTH), as well as endothelium-independent vasodilation (SNP ID), and reduced SBP, DBP, MAP, serum concentrations of TNF-α, IL-6, and endoglin. Carnosine-enriched chicken meat did not adversely affect the lipid profile, renal and liver function, demonstrating its safety in patients with CCS. In conclusion, CCS patients may benefit from carnosine-enriched chicken meat consumption due to enhanced hemodynamic parameters and microvascular vascular relaxation. Furthermore, reduced levels of proinflammatory cytokines may suggest a protective effect of carnosine on the progression of atherosclerosis in CCS patients.

## Figures and Tables

**Table 1:** The effect of consumption of regular and carnosine-enriched chicken meat on anthropometric and hemodynamic parameters in patients with chronic coronary syndrome (CCS).

Parameters	Control		*p* ^a^	Carnosine		*p* ^a^	*p* ^b^
	Before	After		Before	After		
N	19			19		-	-
Age	60.8 (8.8)			60.1 (8.8)		-	-
BMI (kg/m^2^)	28.8 (3.1)	28.8 (3.1)	0.55	32.5 (6.0)	32.3 (6.1)	0.24	0.53
WHR	0.95 [0.92–0.97]	0.94 [0.92–0.97]	>0.99	0.94 (0.04)	0.94 (0.04)	0.08	0.13
SBP (mmHg)	135.4 (10.6)	134.9 (11.6)	0.78	140.6 (12.7)	131.3 (12.2)	**<0.001**	**0.01**
DBP (mmHg)	81.4 (7.6)	78.8 (7.1)	0.07	82.0 [78.0–90.0]	79.2 (5.6)	**0.03**	0.70
MAP (mmHg)	99.3 (8.1)	97.5 (7.5)	0.13	103.1 (8.2)	96.5 (6.1)	**0.001**	**0.04**
HR (beats/min)	59.8 (9.6)	56.5 [51.0–66.3]	0.52	64.8 (8.6)	60.8 (6.3)	**0.003**	**0.02**
SV (ml)	106.6 (16.7)	101.3 (17.9)	0.12	100.9 (23.1)	105.6 (19.2)	0.14	**0.05**
CO (L/min)	6.32 (1.22)	6.04 (1.41)	0.25	6.53 (1.56)	6.45 (1.38)	0.68	0.41
CI (L/min/m^2^)	3.22 (0.60)	3.07 (0.62)	0.24	3.09 (0.56)	3.08 (0.45)	0.90	0.51
SVRI (dyn·s·cm^−5^·m^2^)	2204.0 [2000.0–2494.3]	2444.0 [1947.3–2659.5]	0.90	2456.0 [2023.5–2695.3]	2484.7 (497.8)	0.80	0.92
TACI (mL/m^2^/mmHg)	0.99 (0.23)	0.99 (0.22)	0.76	0.92 (0.16)	0.97 (0.27)	0.33	0.53

**Notes:** Normally distributed data: mean (standard deviation); non-normal: median [interquartile range]. *p* ^a^ ≤ 0.05 (paired *t*-test/Wilcoxon signed-rank); *p* ^b^ ≤ 0.05 (ANCOVA, baseline covariate). **Abbreviations:** N, number of subjects; BMI, body mass index; WHR, waist-to-hip ratio; SBP, systolic blood pressure; DBP, diastolic blood pressure; MAP, mean blood pressure; HR, heart rate; SV, stroke volume; CO, cardiac output; CI, cardiac index; SVRI, systemic vascular resistance index; TACI, total arterial compliance index.

**Table 2:** The effect of consumption of regular and carnosine-enriched chicken meat on the assessment of heart rate variability (HRV) in patients with CCS.

Parameters	Control		*p* ^a^	Carnosine		*p* ^a^	*p* ^b^
	Before	After		Before	After		
Mean RR (ms)	1008.5 (127.7)	974.8 (175.0)	0.23	923.2 (116.1)	972.4 (118.7)	0.08	0.11
SDNN (ms)	49.6 (27.9)	35.9 [29.4–56.1]	0.47	35.2 [31.7–43.9]	33.1 [26.5–54.4]	0.54	0.92
RMS-SD (ms)	25.4 [15.5–47.1]	27.3 [13.9–45.4]	0.62	20.6 [15.6–33.7]	18.7 [15.1–30.1]	0.47	0.92
pNN50 (n.u.)	4.2 [0.4–11.3]	1.8 [0.2–21.3]	0.91	1.6 [0.2–12.0]	1.1 [0.4–8.4]	>0.99	0.67
TI (n.u.)	148.0 [52.7–359.4]	186.8 (127.9)	0.65	173.0 [118.6–221.9]	231.5 (173.2)	0.26	0.26
TP (ln)	6.1 (1.5)	6.0 (1.0)	0.74	6.1 (0.9)	5.9 [5.0–6.7]	0.47	0.91
VLF (ln)	5.4 (1.3)	5.3 (0.8)	0.87	5.5 (0.8)	5.2 (1.3)	0.41	0.70
LF (ln)	5.0 (1.8)	4.7 (1.1)	0.51	4.9 (1.1)	4.7 [3.5–5.7]	0.71	0.88
HF (ln)	4.0 (1.7)	3.9 [2.5–4.6]	0.16	3.6 (1.4)	3.3 [2.8–4.1]	0.54	0.87
LF/HF	1.1 (0.9)	1.0 (1.2)	0.79	1.4 (1.0)	1.2 (0.8)	0.37	0.65

**Notes:** Normally distributed data: mean (standard deviation); non-normal: median [interquartile range]. *p* ^a^ ≤ 0.05 (paired *t*-test/Wilcoxon signed-rank); *p* ^b^ ≤ 0.05 (ANCOVA, baseline covariate). **Abbreviation:** Mean RR, mean value of the distance between two consecutive R waves; SDNN, standard deviation of NN intervals (standard deviation of normal-to-normal interbeat intervals); RMS-SD, root mean square of successive RR interval differences; pNN50, percentage of RR intervals that differ by more than 50 ms; VLF, very low frequency; LF, low frequency; HF, high frequency; LF/HF, ratio of low-frequency power to high-frequency power; TI, triangular index; TP, total power.

**Table 3:** The effect of consumption of regular and carnosine-enriched chicken meat on blood count parameters in patients with CCS.

Parameters	Control		*p* ^a^	Carnosine		*p* ^a^	*p* ^b^
	Before	After		Before	After		
Leukocytes (×10^9^/L)	6.6 [5.8–7.6]	6.7 [6.0–7.2]	0.90	7.2 (1.7)	7.1 (1.7)	0.60	0.72
Erythrocytes (×10^12^/L)	4.9 (0.4)	4.9 (0.4)	0.71	5.1 (0.3)	5.0 (0.3)	0.07	0.67
Hemoglobin (g/L)	143.3 (11.4)	143.2 (12.3)	0.94	147.2 (10.1)	146.2 (8.2)	0.33	0.86
Hematocrit (%)	0.4 (0.0)	0.4 (0.0)	>0.99	0.4 (0.0)	0.4 (0.0)	0.18	0.55
MCV (fL)	88.2 (4.0)	88.6 (3.7)	0.07	87.7 (3.8)	87.9 (3.6)	0.38	0.46
MCH (pg)	29.3 (1.4)	29.3 (1.4)	0.27	29.1 (1.5)	29.6 [28.7–30.1]	**0.005**	0.13
MCHC (g/L)	332.0 [329.0–336.0]	333.0 [326.0–337.5]	0.69	331.7 (8.1)	333.6 (9.3)	0.15	0.17
RDW-CV (%)	13.2 [12.8–13.6]	12.9 [12.7–13.7]	0.48	13.1 [12.8–13.4]	13.2 [12.9–13.3]	0.66	0.86
Platelets (×10^9^/L)	206.4 (43.2)	205.8 (42.6)	0.87	187.0 [169.5–238.0]	215.9 (43.3)	0.10	0.15
MPV (fL)	9.7 (1.9)	10.0 [9.6–10.3]	0.08	9.8 (1.2)	10.0 (0.9)	0.56	0.52

**Notes:** Normally distributed data: mean (standard deviation); non-normal: median [interquartile range]. *p* ^a^ ≤ 0.05 (paired *t*-test/Wilcoxon signed-rank); *p* ^b^ ≤ 0.05 (ANCOVA, baseline covariate). **Abbreviation:** MCV, mean corpuscular volume; MCH, mean corpuscular hemoglobin; MCHC, mean corpuscular hemoglobin concentration; RDW-CV, red cell distribution width (coefficient of variation); MPV, mean platelet volume.

**Table 4:** The effect of consumption of regular and carnosine-enriched chicken meat on biochemical parameters in patients with CCS.

Parameters	Control		*p* ^a^	Carnosine		*p* ^a^	*p* ^b^
	Before	After		Before	After		
Urea (mmol/L)	6.5 (1.7)	6.4 (1.5)	0.82	5.77 (1.39)	5.80 [5.1–7.3]	**0.02**	0.13
Creatinine (µmol/L)	83.9 (19.3)	83.4 (16.2)	0.80	79.0 [70.5–94.5]	86.4 (17.3)	0.39	0.60
Uric acid (µmol/L)	338.5 (89.3)	306.0 [278.5–385.5]	0.93	329.3 (100.1)	324.7 (76.6)	0.72	0.74
Sodium (mmol/L)	141.0 [140.0–141.0]	138.9 (1.5)	**0.01**	140.6 (2.6)	139.7 (2.0)	0.10	0.20
Potassium (mmol/L)	4.3 (0.4)	4.3 (0.3)	>0.99	4.2 (0.3)	4.2 (0.3)	0.88	0.76
Glucose (mmol/L)	6.3 [6.0–6.7]	6.4 [6.0–7.8]	0.26	6.2 [5.8–6.7]	6.3 (1.1)	0.43	0.12
hsCRP (mg/L)	1.3 (1.1)	1.5 (1.1)	0.17	2.2 (3.2)	1.7 (1.3)	0.88	0.91
Calcium (mmol/L)	2.4 (0.1)	2.4 [2.4–2.5]	0.22	2.4 (0.1)	2.4 (0.1)	0.25	0.20
Cholesterol (mmol/L)	3.55 (0.93)	3.52 (0.95)	0.68	3.56 (1.31)	3.45 (1.24)	0.33	0.59
Triglycerides (mmol/L)	1.4 (0.5)	1.5 (1.0)	0.50	1.7 (1.7)	1.5 (1.2)	0.42	0.28
HDL cholesterol (mmol/L)	1.3 (0.4)	1.3 (0.3)	0.45	1.1 (0.2)	1.1 (0.2)	0.23	0.43
LDL cholesterol (mmol/L)	2.3 (0.8)	2.1 (0.8)	0.17	2.3 (1.1)	2.2 (0.9)	0.56	0.48
Total bilirubin (µmol/L)	11.5 (4.6)	10.0 [8.0–14.5]	0.53	11.0 [7.5–13.5]	10.0 [9.0–15.5]	0.17	0.68
AST (U/L)	27.7 (6.1)	29.0 (5.8)	0.27	27.9 (7.5)	28.8 (6.7)	0.38	0.81
ALT (U/L)	32.7 (14.3)	35.3 (18.2)	0.09	33.7 (13.9)	35.7 (15.3)	0.45	0.95

**Notes:** Normally distributed data: mean (standard deviation); non-normal: median [interquartile range]. Bold *p* ^a^ ≤ 0.05 (paired *t*-test/Wilcoxon signed-rank); *p* ^b^ ≤ 0.05 (ANCOVA, baseline covariate). **Abbreviations:** hsCRP, high-sensitivity C-reactive protein; AST, aspartate aminotransferase; ALT, alanine aminotransferase; HDL, high-density lipoprotein; LDL, low-density lipoprotein.

**Table 5:** The effect of consumption of regular and carnosine-enriched chicken meat on serum anti- and proinflammatory cytokines and adhesion molecules in patients with CCS.

Parameters	Control		*p* ^a^	Carnosine		*p* ^a^	*p* ^b^
	Before	After		Before	After		
TGF-1β (pg/mL)	703.8 (229.4)	657.1 (135.9)	0.51	652.9 (207.6)	658.0 (146.4)	0.93	0.97
TNF-α (pg/mL)	60.1 [20.8–126.7]	45.6 [31.6–92.1]	0.47	59.1 [33.2–91.4]	29.2 [18.5–42.8]	**0.04**	0.87
C3a (pg/mL)	13.3 [4.9–28.1]	9.6 [5.9–19.5]	0.54	8.1 [5.9–27.8]	7.6 [5.9–19.6]	0.23	0.54
IFN-γ (pg/mL)	1553 [324–2917]	1287 [789–2482]	0.82	1620 [449–3648]	483.8 [262.2–1204.0]	0.28	0.47
IL-6 (pg/mL)	183.6 [45.7–1071.0]	178.9 [95.6–408.1]	0.12	388.4 [197.9–544.8]	79.1 [40.1–247.1]	**0.007**	0.13
Il-10 (pg/mL)	66.5 [5.3–331.1]	57.9 [17.4–104.6]	0.32	107.7 [78.7–288.2]	23.8 [5.4–171.5]	0.13	0.70
IL-17A (pg/mL)	2.4 [0.8–3.8]	2.7 [1.9–5.4]	0.86	2.3 [1.9–3.7]	2.8 [2.3–4.6]	0.34	0.45
IL-23 (pg/mL)	31.1 (7.8)	32.5 (8.6)	0.54	36.0 (6.4)	35.8 (8.4)	0.96	0.46
ICAM-1 (pg/mL)	2981.3 (2792.0)	2160.4 (1814.7)	0.29	2791.6 (2150.9)	1557.0 (1267.3)	0.08	0.26
VCAM-1 (pg/mL)	6.8 [3.0–16.3]	7.4 [5.0–10.2]	0.83	8.4 [4.2–14.1]	5.4 [3.4–9.5]	0.15	0.92
E-selectin (pg/mL)	407.6 (240.4)	374.3 (173.1)	0.61	476.5 (305.6)	364.7 (226.9)	0.13	0.71
Endoglin (pg/mL)	1.1 [0.4–5.9]	1.7 [0.8–3.8]	0.83	1.3 [0.7–4.7]	0.7 [0.4–1.7]	0.09	**0.04**
MCP-1 (pg/mL)	80.1 [5.0–401.7]	75.8 [18.0–547.6]	0.83	129.0 [29.0–361.3]	68.3 [6.5–217.2]	0.23	0.06

**Notes:** Normally distributed data: mean (standard deviation); non-normal: median [interquartile range]. Bold *p* ^a^ ≤ 0.05 (paired *t*-test/Wilcoxon signed-rank); *p* ^b^ ≤ 0.05 (ANCOVA, baseline covariate). **Abbreviations:** TGF-1β, transforming growth factor β 1; C3a, C3a complement component; TNF-α, tumor necrosis factor alpha; IFN-gamma, interferon gamma; IL-6, interleukin 6; IL-10, interleukin 10; IL-17A, interleukin 17A; IL-23, interleukin 23; ICAM-1, intercellular adhesion molecule 1; VCAM-1, vascular cell adhesion molecule; MCP-1, monocyte chemoattractant protein 1.

## Data Availability

Original data will be shared upon reasonable request to the corresponding author.

## Abbreviations

ACh	Acetylcholine.
ACh: ID	Acetylcholine-induced dilation.
ALT	Alanine transaminase.
AST	Aspartate transaminase.
AUC	Area under the curve.
BMI	Body mass index.
BP	Blood pressure.
CAD	Coronary artery disease.
CCS	Chronic coronary syndrome.
C3a	Complement component 3a.
CI	Cardiac index.
CO	Cardiac output.
FRAP	Ferric-reducing ability of plasma.
HDL	High-density lipoprotein.
HR	Heart rate.
HRV	Heart rate variability.
hsCRP	High-sensitivity C-reactive protein.
ICAM-1	Intercellular adhesion molecule 1.
ICG	Impedance cardiography.
IFN-γ	Interferon gamma.
IL-6	Interleukin-6.
IL-10	Interleukin 10.
IL-17A	Interleukin 17A.
IL-23	Interleukin 23.
LDL	Low-density lipoprotein.
LTH	Local thermal hyperemia.
MAP	Mean blood pressure.
MCP-1	Monocyte chemoattractant protein-1.
MDA	Malondialdehyde.
NO	Nitric oxide.
PORH	Post-occlusive reactive hyperemia.
PU	Perfusion units.
SV	Stroke volume.
SVRI	Systemic vascular resistance index.
SNP	Sodium nitroprusside.
SNP: ID	Sodium nitroprusside-induced dilation.
TACI	Total arterial compliance index.
TBARS	Thiobarbituric acid-reactive substances.
TGF-1β	Transforming growth factor beta 1.
TNF-α	Tumor necrosis factor-alpha.
VCAM-1	Vascular cell adhesion molecule 1.
WHR	Waist-to-hip ratio.
